# The Diagnostic Value of Neutrophil to Lymphocyte Ratio as an Effective Biomarker for Eye Disorders: A Meta-Analysis

**DOI:** 10.1155/2022/5744008

**Published:** 2022-10-15

**Authors:** Mohammad Shirvani, Farhad Soufi, Alireza Nouralishahi, Kimia Vakili, Amirhosseinn Salimi, Brandon Lucke-Wold, Farideh Mousavi, Saman Mohammadzadehsaliani, Shokoufeh Khanzadeh

**Affiliations:** ^1^Geriatric Ophthalmology Research Center, Shahid Sadoughi University of Medical Science, Yazd, Iran; ^2^Poostchi Ophthalmology Research Center, Department of Ophthalmology, School of Medicine, Shiraz University of Medical Sciences, Shiraz, Iran; ^3^Islamic Azad University, Tehran Medical Branch, Tehran, Iran; ^4^Isfahan Eye Research Center, Feiz Hospital, Isfahan University of Medical Sciences, Isfahan, Iran; ^5^Student Research Committee, Faculty of Medicine, Shahid Beheshti University of Medical Sciences, Tehran, Iran; ^6^Student Research Committee, Shahid Sadoughi University of Medical Sciences, Yazd, Iran; ^7^Department of Neurosurgery, University of Florida, Gainesville, USA; ^8^Nikukari Hospital, Tabriz University of Medical Science, Tabriz, Iran; ^9^Student Research Committee, Tabriz University of Medical Sciences, Tabriz, Iran

## Abstract

The neutrophil to lymphocyte ratio (NLR) reflects a dynamic relationship between the innate (neutrophils) and adaptive (lymphocytes) cellular immune response. This systematic review and meta-analysis was conducted to critically evaluate the literature regarding the use of the NLR as a reliable means to detect several ocular disorders. Our study was registered with the PROSPERO (ID: CRD42022314850). Three databases, including PubMed, Embase, Scopus, and the Web of Science, were searched on September 9, 2022, with no restrictions on the article's language. Finally, 32 articles were recognized as eligible for our meta-analysis. We found that patients with eye diseases had significantly elevated levels of NLR in comparison to healthy controls (SMD =0.53, 95% CI =0.35-0.71, *P* < 0.001). In subgroup analysis, patients with keratoconus (SMD =0.69; 95% CI =0.33-1.05, *P* < 0.001), glaucoma (SMD =0.56, 95% CI =0.25-0.87, *P* < 0.001), pterygium (SMD =0.14; 95% CI =0.01-0.26, *P* < 0.001), and idiopathic epiretinal membrane (SMD =0.14; 95% CI =0.01-0.26, *P* < 0.001) had higher levels of NLR compared to healthy controls. However, NLR levels of patients with dry eye disease were similar to healthy controls (SMD =0.32, 95% CI = -0.49-1.13, *P* = 0.435). It can be said that NLR is a valuable marker of systemic inflammation, which is significantly increased in many eye disorders, suggesting that inflammation plays a key role in the pathophysiology of these diseases.

## 1. Introduction

In recent decades, many studies revealed that numerous inflammatory responses are implicated in a variety of eye diseases [1, 2]. Such inflammatory disorders of the eye are one of the most frequent illnesses that cause permanent blindness across the globe. Much of the current literature on the role of inflammation in eye disease focuses on simple hematological biomarkers such as neutrophil to lymphocyte ratio (NLR) and platelet to lymphocyte ratio (PLR) due to their low cost and accessibility [3–31]. NLR reflects online dynamic relationship between the adaptive (lymphocytes) and innate (neutrophils) cellular immune response. The diagnostic and prognostic value of NLR as an affordable, novel, and widely accepted marker has also been discussed in several human disorders including eye diseases such as glaucoma, dry eye disease (DED), idiopathic epiretinal membrane (iERM), retinal vein occlusion, keratoconus (KC), pterygium, and diabetic retinopathy [3–35]. This ratio is critical to early detection as a lot of patients with eye diseases were previously healthy and asymptomatic.

KC is an ectatic corneal condition that causes myopia and irregular astigmatism, and leads to vision loss due to stromal scarring, protrusion, and thinning in the cornea. Systemic inflammatory indicators such as PLR, monocyte/high-density lipoprotein cholesterol ratio, and red blood cell distribution width have also been demonstrated to be higher in individuals with KC [5, 11, 13, 15]. However, to date, there has been little agreement on the importance of NLR level in these patients [5, 11, 13, 15, 23, 27].

Glaucoma is a neurodegenerative disease that causes progressive atrophy of the optic disc leading to visual field defects. This disorder is often linked with high intraocular pressure (IOP), which is an established risk factor for disease development and permanent blindness [36]. In the literature focused on glaucoma, the relative importance of NLR has been subject to debate, because some studies reported significant differences in NLR levels between glaucoma patients and healthy control patients [3, 4, 9, 14, 18, 20, 25, 26, 29, 31, 37, 38].

Pterygium is a fibrovascular tissue growth on the cornea that leads to persistent irritation in the eye and astigmatism [39]. Recently, the literature has emerged that offers contradictory findings about the NLR level in pterygium patients compared to healthy individuals [12, 16, 17, 19, 22, 35].

Dry eye disease or DED is characterized by the symptoms such as foreign body sensation, discharge, and even obscured vision. The most updated classification subdivides DED into two types: tear-deficient and evaporative DED. In the tear-deficient DED subtype, malfunctioning lacrimal glands are often diagnosed, and this deficiency is strongly associated with an autoimmune response that may target the body's salivary and lacrimal glands (Sjögren's syndrome). Many studies have shown increased amounts of proinflammatory mediators such as interleukin (IL)-1, IL-6, and tumor necrosis factor-alpha (TNF-*α*) in tear fluid of DED patients [6]. With respect to NLR level in DED, some studies reported that NLR level is higher in DED in comparison to healthy controls [21, 24, 28]. Vice versa, one study reported different results [6].

In addition, iERM is a relatively prevalent macular disorder among older people due to an abnormal vitreomacular interface [40]. It may cause decreased visual acuity, metamorphopsia, monocular diplopia, macropsia, and micropsia [40]. Several researchers have reported that NLR levels were higher in iERM patients than healthy controls [7, 8, 10, 30].

Eye disorders are characterized by some degree of inflammatory burden [41]. On the other hand, NLR is associated with increased inflammation in various conditions such as type 2 DM [42], autoimmune conditions [43], stroke [44, 45], thyroid disorders [46], functional bowel disease [47], and even COVID-19 infection [48]. In addition, there has been an increase in the number of papers related to the role of NLR in several eye diseases [3–31, 35, 37, 38], and it has gained prominence as an early predictive marker for several eye diseases that were mentioned earlier. However, much uncertainty still exists about this relationship, because most studies have only been carried out on a small sample size. In addition, the literature has emerged that offers inconsistent findings about these interesting topics. Existing accounts fail to resolve these discrepancies since much of the research up to now has been original except in the case of retinal vein occlusion [49], age-related macular degeneration [50], and diabetic retinopathy [51]. No meta-analysis has been conducted in this regard [25]. So, a critical review of the available literature has yet to be performed regarding these important topics. This paper seeks to remedy these problems by reviewing the studies on the prognostic and diagnostic value of NLR in several ocular disorders, including KC, glaucoma, pterygium, iERM, and DED. The key is to understand what an elevated ratio might mean for a patient with eye disease to help clinicians institute early interventions and improve outcomes.

## 2. Methods

This study was conducted in accordance with the Meta-Analysis of Observational Studies in Epidemiology (MOOSE) guideline and the Preferred Reporting Items for Systematic Reviews and Meta-Analyses (PRISMA). Our study was registered with the PROSPERO (ID: CRD42022314850).

### 2.1. Search Strategy

Three databases, including PubMed, Embase, Scopus, and the Web of Science, were searched up to September 9, 2022. In our literature search, we included a combination of keywords, such as NLR, neutrophil to lymphocyte ratio, eye disease, and ophthalmology, in the form of title/abstract words or medical subject headings. For details, please refer to supplementary appendix A (available here).

### 2.2. Study Selection

After eliminating the duplicates, one author assessed the title and abstract of the remaining articles to exclude obviously unrelated reports. The complete text of the remaining references was then separately checked for eligibility by two authors. Any other relevant studies were found in the reference lists of recognized articles. If there was a disagreement, a third author would be brought in to debate the situation and establish a consensus.

We identify eligible studies according to the PICOS (population, intervention, control, outcomes, and study design) principle in order to ensure the systematic search of available literature. The inclusion criteria were presented below:

- Population. Patients with KC, glaucoma, pterygium, iERM, or DED
- Intervention. NLR
- Control. Healthy controls
- Outcomes. The diagnostic performance of NLR in eye diseases
- Study Design. We expected papers to be case-control or cross-sectional. However, we did not limit our search to any particular research design

Review articles, letters to editors, animal studies, single case reports, and studies presented as conference abstracts were not considered eligible. In addition, we excluded studies on the relationship between NLR and retinal vein occlusion, age-related macular degeneration, and diabetic retinopathy, because the relevant meta-analysis in these contexts was published.

### 2.3. Data Extraction and Quality Assessment

The first author's name, year of publication, language, study location, ethnicity, study design, eye disease type, number of cases and controls, and NLR level data in cases and controls were all collected. The medication of the patients with eye disorders could potentially conceal the actual association of NLR levels with eye disorders; so the exclusion criteria based on medication use in the included studies were extracted as well.

We used the ROBINS-1 (formerly called A Cochrane Risk of Bias Assessment Tool) for assessing the quality of included studies [52].

### 2.4. Publication Bias and Statistical Analysis

The difference in means in NLR between patients and healthy controls was the primary outcome; thus, we used a quantitative synthesis to compute the difference in NLR means between two groups (meta-analysis). The difference in NLR between patients with different clinical subtypes of glaucoma and healthy controls was the secondary outcome; thus, subgroup meta-analyses for patients with primary open-angle glaucoma (POAG), secondary open-angle glaucoma (SOAG), primary closed angle glaucoma (PCAG), and secondary closed angle glaucoma (SCAG) were performed. In addition, we conducted a subgroup meta-analysis based on research location on the connection between NLR and glaucoma. STATA 12.0 was used to conduct the meta-analyses (Stata Corporation, College Station, TX, USA). When mean and standard deviation (SD) were not supplied, median and interquartile ranges were utilized to determine mean and SD using Wan, X. et al. method [53]. Because of the presumed heterogeneity across the studies due to diverse study designs, methods, and populations, a random-effects model was adopted. Cochran's *Q* and *I*^2^ were used to determine the level of heterogeneity. A Funnel plot was used to assess publication bias. Forest plots were used to show the summary measures.

## 3. Results

### 3.1. Literature Search and Selection

**Figure 1:**
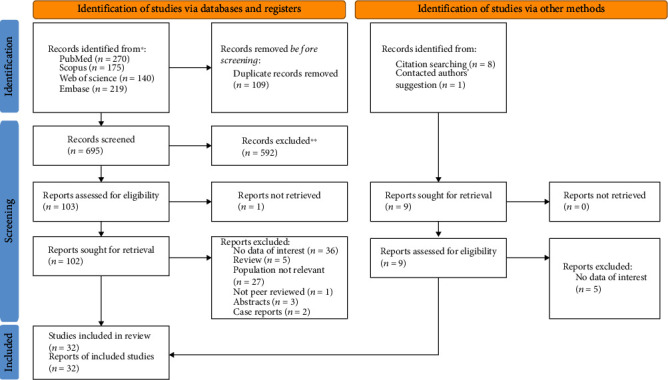
PRISMA 2020 flow diagram for new systematic reviews which includes searches of databases, registers, and other sources.

A total of 813 records were retrieved in the database search and manual search of citation list of articles. After the exclusion of duplicates, 32 studies [3–31, 35, 37, 38] were included in the systematic review and meta-analysis. The process of inclusion and exclusion is detailed in the PRISMA flow diagram, provided in Figure 1.

### 3.2. Characteristics of the Included Studies

Of 32 studies included in this meta-analysis, 26 studies [3–19, 22–28, 30, 35] were conducted in Turkey, four in China [20, 21, 29, 31], one study in India [37], and one in Korea [38]. Concerning document language, 31 studies were in English [3–31, 37, 38], and one study in Turkish [35]. In terms of study design, there were 11 prospective [4–6, 11, 13, 16, 21, 24, 27, 28, 30] and 21 retrospective studies [3, 7–10, 12, 14, 15, 17–20, 22, 23, 25, 26, 29, 31, 35, 37, 38]. Overall, 3242 healthy controls and 3378 patients with eye diseases were enrolled in the selected studies. The general characteristics of the selected studies and their quality score are presented in Table 1. We found six studies on KC [5, 11, 13, 15, 23, 27], six studies on pterygium [12, 16, 17, 19, 22, 35], four studies on DED [6, 21, 24, 28], and four studies on iERM [7, 8, 10, 30]. Also, the association between NLR and glaucoma was investigated in 12 studies [3, 4, 9, 14, 18, 20, 25, 26, 29, 31, 37, 38], of which four were conducted among East Asian patients [20, 29, 31, 38] and eight among Caucasian patients [3, 4, 9, 14, 18, 25, 26, 37]. Among these ten studies, we found five studies on POAG [3, 4, 14, 25, 29], six studies on SOAG [3, 9, 18, 26, 37, 38], two studies on PCAG [14, 20], and one study on SCAG [31].

Of 32 studies, 23 studies [4, 6, 7, 10–13, 15–17, 19, 21–24, 26–30, 35, 38] excluded the patients who were smoking, using alcohol, or receiving medications that could affect the ocular surface of the eye and blood parameters. These include systemic or ocular medications including topical steroids, anti-inflammatory medications, iron preparations, vitamins, and chemotherapeutic agents. Remaining studies did not declare any exclusion criteria based on the medication taking history of the patients. However, they mentioned that excluded patients with systematic disorders such as diabetes mellitus, cardiovascular diseases, arterial hypertension, chronic obstructive lung disease, malignancies, renal dysfunction, liver dysfunction, hematologic or autoimmune disorders, and chronic systemic inflammatory disorders. It can imply the exclusion of patients with a history of receiving medications with systematic effects. With these strict exclusion criteria, the effect of medication use on blood parameters was modified in included studies.

Table 2 shows the results of the publication bias and heterogeneity tests in every single outcome (KC, glaucoma, pterygium, iERM, or DED).

### 3.3. The Association between NLR Levels and Overall Risk of Eye Diseases

**Figure 2:**
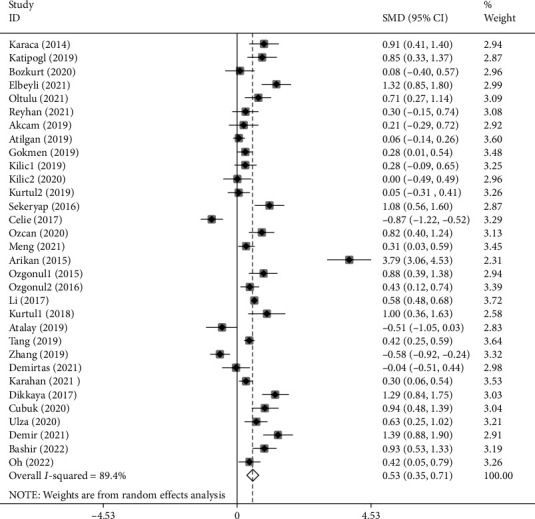
Meta-analysis of differences in NLR levels between patients with eye diseases and healthy controls (*P* value<0.001).

Overall, 3323 healthy controls and 3558 patients with several eye diseases were compared in terms of NLR levels in 32 studies [3–31, 35, 37, 38]. Patients with eye diseases had significantly higher levels of NLR in comparison to healthy controls (SMD =0.53, 95% CI =0.35-0.71, *P* < 0.001) (Figure 2).

### 3.4. Keratoconus and NLR

**Figure 3:**
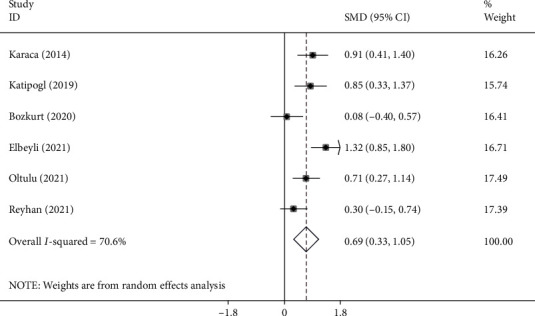
Meta-analysis of differences in NLR levels between KC patients and healthy controls (*P* value<0.001).

NLR levels in keratoconus patients were compared with those of healthy controls in six studies [5, 11, 13, 15, 23, 27] with 245 patients with keratoconus and 211 healthy controls. Compared with the control group, the keratoconus patients' NLR levels were significantly higher (SMD =0.69; 95% CI =0.33-1.05, *P* < 0.001) (Figure 3).

### 3.5. Dry Eye and NLR

**Figure 4:**
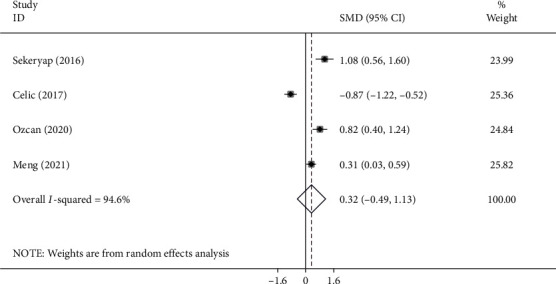
Meta-analysis of differences in NLR levels between DED patients and healthy controls (*P* value =0.435).

Four studies [6, 12, 16, 17, 19, 21, 22, 24, 28, 35] including 262 patients and 236 healthy controls investigated the NLR level differences between dry eye patients and healthy controls. The pooled results showed that there were no significant differences between DED patients and healthy individuals in NLR level (SMD =0.32, -0.49-1.13, *P* = 0.435) (Figure 4).

### 3.6. Pterygium and NLR

**Figure 5:**
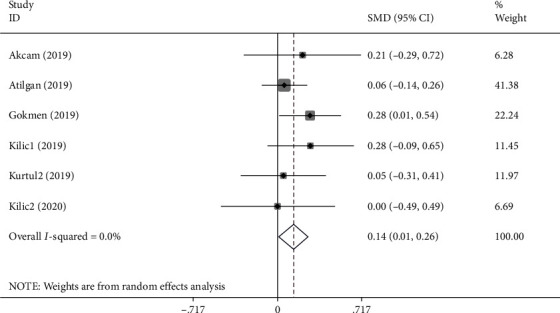
Meta-analysis of differences in NLR levels between pterygium patients and healthy controls (*P* value =0.033).

Pterygium patients' NLR levels were compared with those of healthy controls in six studies [12, 16, 17, 19, 22, 35] including 1384 patients and 1238 controls. Compared to healthy individuals, patients with pterygium had significantly higher levels of NLR (SMD =0.14; 95% CI =0.01-0.26, *P* < 0.001) (Figure 5).

### 3.7. Idiopathic Epiretinal Membrane and NLR

**Figure 6:**
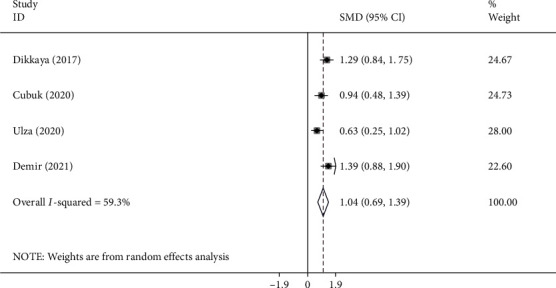
Meta-analysis of differences in NLR levels between iERM patients and healthy controls (*P* value<0.001).

In four studies [7, 8, 10, 30], iERM patients' NLR levels were compared with those of healthy controls including 178 patients and 176 controls. Compared to healthy individuals, patients with iERM had significantly higher levels of NLR (SMD =0.14; 95% CI =0.01-0.26, *P* < 0.001) (Figure 6).

### 3.8. Glaucoma and NLR

**Figure 7:**
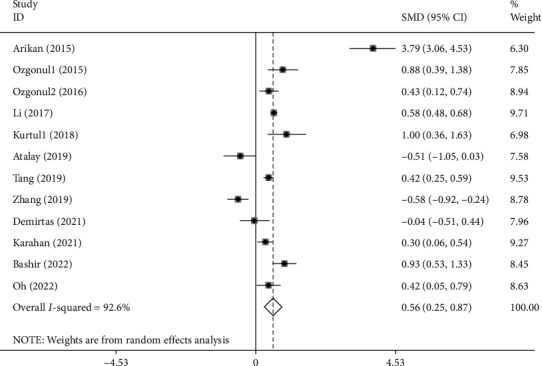
Meta-analysis of differences in NLR levels between glaucoma patients and healthy controls (*P* value<0.001).

The association between NLR and glaucoma was investigated in 12 studies [3, 4, 9, 14, 18, 20, 25, 26, 29, 31, 37, 38] including 1568 glaucoma patients and 1737 healthy controls. NLR levels were significantly higher in glaucoma patients compared with controls (SMD =0.56; 95% CI =0.25-0.87, *P* < 0.001) (Figure 7).

**Figure 8:**
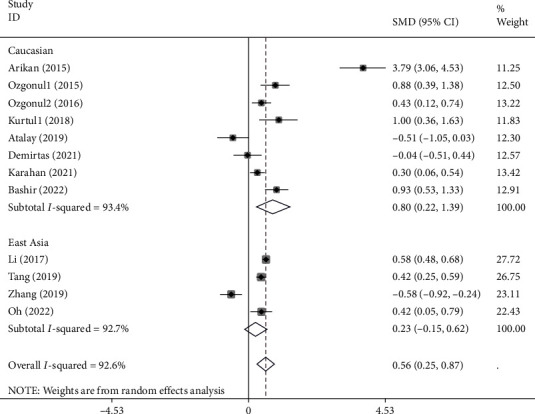
Subgroup analysis of the differences in NLR levels between glaucoma patients and healthy controls according to ethnicity.

In subgroup analysis according to ethnicity, there were four studies including East Asian patients [20, 29, 31, 38], consisting of 1111 patients and 1234 controls, and eight studies including Caucasian patients [3, 4, 9, 14, 18, 25, 26, 37] including 457 patients and 483 controls. The pooled results showed that the NLR levels in Caucasian patients with glaucoma were significantly more than healthy controls (SMD =0.80, 95% CI =022-1.39, *P* value<0.001). However, the NLR levels of East Asian patients were similar to those of healthy controls (SMD =0.23, 95% CI = -0.15-0.62, *P* = 0.03) (Figure 8).

**Figure 9:**
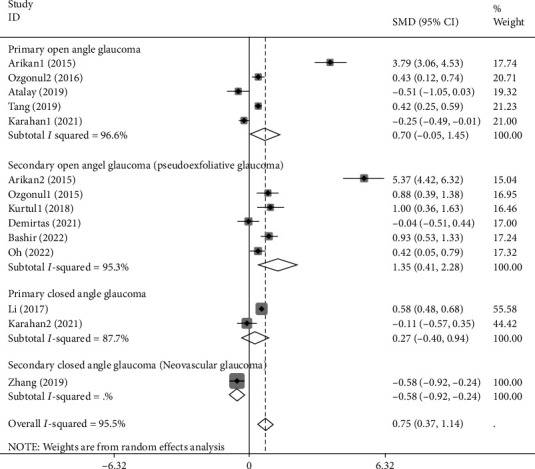
Subgroup analysis of the differences in NLR levels between glaucoma patients and healthy controls according to the glaucoma type.

In the next step, we categorized studies in four groups according to the type of patients' glaucoma and conducted the second subgroup intending to comparing glaucoma patients and healthy controls in each group. There were five studies on primary open-angle glaucoma [3, 4, 14, 25, 29] including 595 patients and 547 controls, six studies on secondary open-angle glaucoma [3, 9, 18, 26, 37, 38] comprising 186 patients and 376 controls, two studies on primary closed angle glaucoma [14, 20] with 793 patients and 870 controls, and one study on secondary closed angle glaucoma [31] with 59 patients and 84 controls. NLR was significantly higher in patients with SOAG (SMD =1.35, 95% CI =0.41-2.28, *P* = 0.005) and significantly lower in patients with SCAG (SMD = -0.58, 95% CI = -0.9 - -0.24, *P* = 0.42), compared to healthy controls. However, when focusing on the differences between patients with POAG and PCAG compared to healthy controls, we found no differences (SMD =0.70, 95% CI = -0.05-1.45, *P* = 0.06 and SMD =0.27, 95% CI = -0.40-0.94, *P* = 0.001, respectively) (Figure 9).

### 3.9. Publication Bias

**Figure 10:**
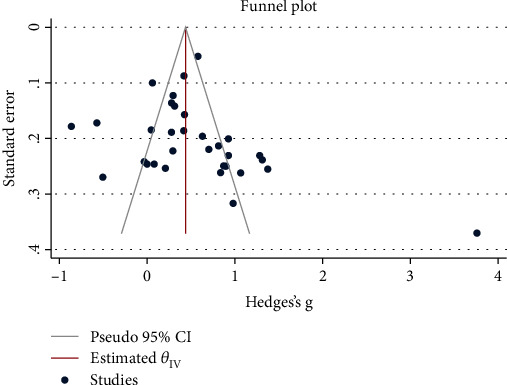
Funnel plot assessing publication bias across studies on NLR level in patients with eye diseases.

As presented in Figure 10, the results of studies on difference in NLR levels between patients with eye diseases and healthy controls showed no significant publication bias.

## 4. Discussion

In this systematic review and meta-analysis, we compared NLR between healthy controls and patients with a variety of eye diseases, including keratoconus, glaucoma, pterygium, iERM, and DED, to see if this marker is sensitive enough for the estimation of the severity of systemic inflammation in these patients. We found that except for patients with eye disorders, NLR levels were significantly higher in patients with these disorders than healthy controls, implying the critical role of inflammation in developing these disorders.

Neutrophils and lymphocytes are key immune system cellular components. Neutrophils are a type of innate immunity cell that can produce chemokines, cytokines, vascular endothelial growth factor, and matrix metalloproteinase to reinforce the initial line of immune system. Lymphocytes, which are adaptive immunity cells, are also fine controllers of particular immune responses [50]. As neutrophils and lymphocytes can interact with each other, their ratio and sheer numbers have an impact on the immune response's amplitude. Increased neutrophil numbers, in particular, decrease lymphocyte activity [54]. Recently, NLR has emerged as an indicator of systemic inflammation in a variety of disorders including eye diseases, and it has been used as an independent prognostic biomarker in various clinical setting, predicting major mortality, morbidity, and long-term survival [51, 55–58].

NLR was significantly higher in patients with KC compared to healthy controls. According to previous studies, proinflammatory cytokines (such as TNF-*α*, IL-6, and matrix metalloproteinase) levels are considerably greater in tear fluid of KC patients [11, 59]. Degradation of the corneal extracellular matrix and alteration of its cellular components may occur as a result of oxidative stress and inflammation [59–62]. There are also further reports that showed immunohistochemically evidence of inflammation in the keratoconic cornea, including leukocyte deposition, macrophage infiltration, and dendritic Langerhans cell abundance [63]. Loh et al. also investigated the cytokine profile of human keratoconic corneas. They agreed with the past evidence implicating inflammatory activation in KC and suggested that KC could be reclassified as a chronic inflammatory corneal disorder [64]. A meta-analysis by Zhang et al. revealed that tear levels of proinflammatory cytokines including IL-1, IL-6, and TNF-*α* were elevated in KC patients compared to healthy controls, suggesting that the cytokine profile is definitely altered in these patients and inflammation implicates in the pathophysiology and progression of the disease [65]. Karaca et al. studied the relationship between NLR and KC and found that NLR levels were greater in progressive patients with KC in comparison to nonprogressive patients [13]. In their research, they discovered a significant positive link between NLR and progression of the disease. Apart from NLR, systemic immune-inflammation index (SII) values were found to be considerably higher in the KC group in a study by Elbeyli et al. Furthermore, they observed that SII levels steadily increased in the severe KC subgroup [11].

In the second analysis, we found that NLR was significantly higher in patients with glaucoma compared to healthy controls. In a subgroup analysis according to the study location, NLR was significantly higher in Caucasian patients with glaucoma compared to healthy controls. However, it was not different between East Asian patients and controls. In a subgroup analysis according to the glaucoma type, NLR was significantly higher only in SOAG group compared to healthy controls. Glaucoma is a collection of progressive visual neuropathic disorders that is estimated to be one of the leading causes of permanent blindness globally [66]. While IOP is a well-established and modifiable risk factor, the actual mechanism of both POAG and PCAG is still being debated [66, 67]. Among the underlying molecular mechanisms, autoimmune processes, vascular dysfunction, oxidative stress, and inflammatory responses are the most important ones [14]. As a result, systemic inflammation may play a role in the pathophysiology of glaucoma.

Our results showed that NLR levels in patients with pterygium were higher than healthy controls. Exposure to ultraviolet irradiation and low moisture are the most prevalent recognized predisposing factors for pterygium. Aside from these factors, recent data reveals that local oxidative stress, as well as local inflammatory mediators, has a role in the initiation and growth of pterygial tissue [68, 69]. However, unlike local inflammation, the literature on the systemic inflammatory state of pterygium patients is sparse, and there is no clear agreement on the correlation between NLR and pterygium. These findings suggest that the local inflammatory response, rather than the systemic inflammation, is considerably more active in the pathophysiology of primary pterygium. However, in our meta-analysis, we found a significantly increased NLR in pterygium patients compared to healthy controls, which may imply to the fact that systemic inflammation is also correlated with incidence of pterygium.

In addition, we showed that NLR was not different between DED patients and healthy controls. The lipid layer of the tear film, which regulates the evaporation process, controls the wettability of the ocular surface. Because of the excessive evaporation and unstable lipid layer in DED, the osmolarity of tear fluid rises and therefore the release of proinflammatory cytokines is stimulated by the hyperosmotic tear fluid [70]. So, DED has been linked to increased levels of proinflammatory cytokines such as different interleukins (IL-1, IL-2, IL-6, and IL-8), TNF, transforming growth factor, and matrix metallopeptidase [71, 72]. From the many cellular components of the immune response participating in DED, lymphocytes constitute one crucial component, especially in tear-deficient type. However, in our study, the data did not show any significant difference between patients with DED and healthy controls, which may show that this marker is not sensitive enough for dry eye condition when it is evaluated in larger populations.

In addition, we found that iERM patients had elevated levels of NLR in comparison to healthy controls. In accordance with the present result, previous studies have demonstrated that vitreous of iERM patients had elevated levels of several cytokines such as vascular endothelial growth factor, nerve growth factor, fibroblast growth factor, and compared with that of healthy controls [73]. It seems possible that these results are due to the fact that local and systematic inflammations have an important role in iERM development.

### 4.1. Clinical Utility of the Results

NLR is a measure that is readily obtained on admission from a white blood cell differential and is associated with no additional cost or labor. It shows balance between innate (neutrophil) and the adaptive (lymphocyte) immune system [74]. Recent studies show that NLR can predict eye disorders with relatively high sensitivity and specificity. As evidenced by these results, restoring balance between the innate and adaptive immune system may serve as attractive therapeutic targets; so medications aimed at reducing NLR may be efficacious for treating and even preventing such disorders. Theoretically, reduction in NLR values could be used to measure therapeutic efficacy, reflecting restoration of balance within these systems. Further, our findings support NLR to be a promising biomarker that can be readily integrated into clinical settings to aid in the prediction and prevention of eye disorders. Ultimately, with the development of new biomarkers and therapeutic modalities, we can better prevent and treat eye disorders to decrease long-term morbidity and mortality.

### 4.2. Limitations

The findings of this report are subject to at least four limitations. Small sample size of included studies was the first major limitation. Second, the majority of them were retrospective. Thirdly, the studies did not evaluate these patients' NLR levels obtained from tear, due to limited number of studies. Fourthly, there were a limited number of studies on the role of NLR in DED and iERM. Meanwhile, several questions remain unanswered at present on the association between NLR and many other eye diseases, due to the lack of published papers on them. So, there is abundant room for further progress in determining this association. In addition, the majority of studies were conducted in China and Turkey; so further work is required to establish this association. Nonetheless, there were three main strengths in the present review. First, the present study, to our best knowledge, serves as the first meta-analysis exploring the correlation between NLR and eye diseases. Second, the studies were included in the final analysis based on clear inclusion and exclusion criteria. Third, our systematic search, in conjunction with a manual review of references from resulting articles without any limitation on language or date, has ensured a thorough and reliable search of literature and serves as a notable strength of this study.

## 5. Conclusion

In summary, it can be said that NLR is a valuable marker of systemic inflammation, which is significantly increased in many eye disorders including KC, glaucoma, pterygium, and iERM, but not DED, suggesting that inflammation plays a key role in the pathophysiology of these disease.

## Figures and Tables

**Table 1:** Characteristic of included studies.

Author	Year	Country	Exclusion criteria based on medication taking history	Patients with eye diseases			Healthy controls			Quality
				Number	NLR		Number	NLR		
					Mean	SD		Mean	SD	
Keratoconus										
Karaca	2014	Turkey	Smoking habit, current anti-inflammatory therapies	54	2.59	0.89	25	1.86	0.52	Moderate
Katipoglu	2019	Turkey	Anti-hyperlipidemic therapy or steroid use, or smoking and alcohol use	31	2.30	0.80	31	1.70	0.60	Low
Bozkurt	2020	Turkey	ND	35	2.01	0.53	30	1.97	0.41	Moderate
Elbeyli	2021	Turkey	Anti-hyperlipidemic therapy or steroid use, or smoking and alcohol use, current anti-inflammatory therapies	42	2.50	0.80	42	1.70	0.30	Low
Oltutu	2021	Turkey	Smoker, and alcohol abuse, any medication that could affect blood parameters	43	2.30	0.87	43	1.77	0.61	Critical
Reyhan	2021	Turkey	Currently receiving anti-inflammatory drugs, smoking, and alcohol use	40	2.15	1.46	40	1.81	0.72	Moderate
Dry eye disease										
Sekeryap	2016	Turkey	Smoking, taking anti-inflammatory drugs	33	2.80	1.40	32	1.60	0.70	Low
Celic	2017	Turkey	Smoking, receiving ocular/systemic drug	78	1.84	0.50	60	2.60	1.20	Moderate
Ozcan	2020	Turkey	Smoking, systemic or ocular medications including topical steroids (the previous use during at least 3 months) and anti-inflammatory medications, that could affect the ocular surface of the eye and blood parameters	47	2.26	0.55	47	1.81	0.55	Moderate
Meng	2021	China	Receiving hormone medication and systemic or tropical immunosuppressant during three months	104	2.59	1.25	97	2.20	1.24	Moderate
Pterygium										
Akcam	2019	Turkey	Receiving topical/systemic drug, cigarette/alcohol using	30	1.86	0.38	31	1.76	0.54	Moderate
Atilgan	2019	Turkey	Steroid use	200	2.10	0.89	200	2.05	0.80	Low
Gokmen	2019	Turkey	Smoking, using steroid, or oral contraceptive drugs	111	2.53	2.27	106	2.04	1.03	Low
Kilic 1	2019	Turkey	Receiving antioxidant or anti-inflammatory medications or any topical or systemic drugs	71	1.90	0.59	46	1.73	0.67	Moderate
Kurtul2	2019	Turkey	Receiving immunosuppressive treatment	61	1.85	0.82	55	2.72	0.79	Low
Kilic 2	2020	Turkey	Past systemic medical therapy, smoking	35	2.72	3.61	30	1.81	3.97	Low
Idiopathic epiretinal membrane										
Dilkaya	2017	Turkey	Any special drug use (e.g., corticosteroids iron preparations, vitamins, and chemotherapeutic agents)	43	3.03	1.20	46	1.77	0.70	Moderate
Cubuk	2020	Turkey	Any drug use	42	2.85	0.72	40	2.18	0.71	Moderate
Ulza	2020	Turkey	A history of systemic drug use	57	2.10	0.90	51	1.64	0.46	Low
Demir	2021	Turkey	Receiving medications affecting whole blood parameters such as corticosteroid and iron and chemotherapeutic	36	2.13	0.43	39	1.63	0.28	Low
Glaucoma										
Arikan	2015	Turkey	ND	40	2.30	0.20	40	1.70	0.10	Low
Ozgonul1	2015	Turkey	ND	29	2.45	0.82	42	1.84	0.59	Low
Ozgonul2	2016	Turkey	Any special drug use (e.g., corticosteroids, iron preparations, vitamins, and chemotherapeutic agents)	84	2.33	0.90	80	1.98	0.73	Moderate
Li	2017	China	ND	771	2.85	1.94	770	1.98	0.86	Serious
Kurtul 1	2018	Turkey	ND	14	2.19	0.78	43	1.56	0.58	Moderate
Atalay	2019	Turkey	Smoking	28	1.82	0.68	27	2.21	0.84	Moderate
Tang	2019	China	Any special drug use (e.g., corticosteroids, iron preparations, vitamins, and chemotherapeutic agents)	240	2.59	1.40	300	2.08	1.05	Moderate
Zhang	2019	China	ND	59	2.07	0.88	84	2.73	1.30	Low
Demirtas	2021	Turkey	ND	22	3.82	4.28	71	4.04	6.47	Serious
Karahan	2021	Turkey	ND	200	5.40	9.28	100	3.00	4.91	Moderate
Bashir	2022	India	ND	40	2.06	0.48	80	1.49	0.67	Moderate
Oh	2022	Korea	History of ocular drug use except cataracts	41	1.94	0.60	100	1.70	0.56	Moderate

NLR: neutrophil to lymphocyte ratio; SD: standard deviation; ND: not declared.

**Table 2:** The results of the publication bias and heterogeneity tests.

Outcome	Number of studies	SMD(95% CI)	Heterogeneity		Publication bias	
			*I* ^2^ statistics	Q test *P* value	Egger's test *P* value	Begg's test *P* value
Keratoconus	6	0.69 (0.33-1.05)	70.6%	0.004	0.65	1.00
Dry eye disease	4	0.32 (-0.49-1.13)	94.6%	<0.001	0.30	0.30
Pterygium	6	0.14 (0.01-0.26)	0.0%	0.727	0.75	1.00
Idiopathic epiretinal membrane	4	0.14 (0.01-0.26)	59.3%	0.061	0.01	0.08
Glaucoma	12	0.56 (0.25-0.87)	92%	<0.001	0.06	0.53

## Data Availability

All data generated or analyzed during this study are included in this published article.

## Abbreviations

NLR: Neutrophil to lymphocyte ratio
PLR: Platelet to lymphocyte ratio
DED: Dry eye disease
iERM: Idiopathic epiretinal membrane
KC: Keratoconus
IOP: Intraocular pressure
IL: Interleukin
TNF-*α*: Tumor necrosis factor-alpha
MOOSE: Meta-Analysis of Observational Studies in Epidemiology
PRISMA: Preferred Reporting Items for Systematic Reviews and Meta-analyses
NOS: Newcastle-Ottawa scale
POAG: Primary open-angle glaucoma
SOAG: Secondary open-angle glaucoma
PCAG: Primary closed angle glaucoma
SCAG: Secondary closed angle glaucoma
SD: Standard deviation
SII: Systemic immune-inflammation index.

## Supplementary Materials

Supplementary Materials supplementary appendix A shows the exact search strategy in all databases, searched in our study.
