# Supplementary material for: The Diagnostic Value of Neutrophil to Lymphocyte Ratio as an Effective Biomarker for Eye Disorders: A Meta-Analysis
Source: Biomed Res Int. 2022 Oct 15;2022:5744008. doi: 10.1155/2022/5744008 (PMC9587911; doi:10.1155/2022/5744008)
Supplement: Supplementary Materials — supplementary appendix A shows the exact search strategy in all databases, searched in our study. [file 5744008.f1.docx]

**Search strategy**

**PubMed**

("eye diseases"[MeSH Terms] OR "Ophthalmology"[Title/Abstract] OR "Ophthalmology"[MeSH Terms] OR (("ocular"[Title/Abstract] OR "eye"[Title/Abstract] OR "eye"[MeSH Terms]) AND ("disease"[MeSH Terms] OR "disease"[Title/Abstract] OR "disorder"[Title/Abstract]))) AND ("NLR"[Title/Abstract] OR "neutrophil to lymphocyte ratio"[Title/Abstract] OR "neutrophil lymphocyte ratio"[Title/Abstract])

-----------------------------------------------------------------------------

**Scopus**

( TITLE-ABS-KEY ( nlr  OR  "neutrophil to lymphocyte ratio"  OR  "neutrophil-lymphocyte ratio" ) )  AND  ( ( ( TITLE-ABS-KEY ( disease  OR  disorder ) )  AND  ( TITLE-ABS-KEY ( ocular  OR  eye ) ) )  OR  ( TITLE-ABS-KEY ( ophthalmology ) ) )

-------------------------------------------------------------------------------

**Web of science**

ALL=(nlr OR neutrophil to lymphocyte ratio OR neutrophil-lymphocyte ratio) AND ALL=(((disease OR disorder ) AND ( ocular OR eye )) OR ophthalmology )

-------------------------------------------------------------------

Embase

( NLR OR "neutrophil to lymphocyte ratio" OR "neutrophil-lymphocyte ratio" ) AND ( ( ( disease OR disorder ) AND ( ocular OR eye ) ) OR ophthalmology )
